# RETRACTION: A Meta‐Analysis Evaluating Wound Infections and Other Complications following Distal versus Complete Gastrectomy for Gastric Cancer

**DOI:** 10.1111/iwj.70509

**Published:** 2025-04-18

## Abstract

**RETRACTION:** ImamM. S. , Abdel‐SattarR. M. , AlotaibiG. R. , AlotaibiK. S. , AlmuthaybiriN. M. , AlshahraniS. A. , AlghamdiM. A. , and AbdelrahimM. E. A. , “A Meta‐Analysis Evaluating Wound Infections and Other Complications following Distal versus Complete Gastrectomy for Gastric Cancer,” International Wound Journal 21, no. 4 (2024): e14516, 10.1111/iwj.14516.38084020 PMC10958092

The above article, published online on 11 December 2023, in Wiley Online Library (http://onlinelibrary.wiley.com/), has been retracted by agreement between the journal Editor in Chief, Professor Keith Harding; and John Wiley & Sons Ltd. Following an investigation by the publisher, all parties have concluded that this article was accepted solely on the basis of a compromised peer review process. In addition, the investigation found significant textual overlap between this article and another article by different authors [1]. The editors have therefore decided to retract the article. The authors disagree with the retraction.

Reference

[1] LiZ. , BaiB. , XieF. , ZhaoQ. , “Distal Versus Total Gastrectomy for Middle and Lower‐Third Gastric Cancer: A Systematic Review and Meta‐Analysis,” International Journal of Surgery 53 (2018): 163–170, 10.1016/j.ijsu.2018.03.047.29602012

**RETRACTION:** M. S. Imam , R. M. Abdel‐Sattar , G. R. Alotaibi , K. S. Alotaibi , N. M. Almuthaybiri , S. A. Alshahrani , M. A. Alghamdi , and M. E. A. Abdelrahim , “A Meta‐Analysis Evaluating Wound Infections and Other Complications following Distal versus Complete Gastrectomy for Gastric Cancer,” International Wound Journal 21, no. 4 (2024): e14516, 10.1111/iwj.14516.38084020 PMC10958092

The above article, published online on 11 December 2023, in Wiley Online Library (http://onlinelibrary.wiley.com/), has been retracted by agreement between the journal Editor in Chief, Professor Keith Harding; and John Wiley & Sons Ltd. Following an investigation by the publisher, all parties have concluded that this article was accepted solely on the basis of a compromised peer review process. In addition, the investigation found significant textual overlap between this article and another article by different authors [1]. The editors have therefore decided to retract the article. The authors disagree with the retraction.

**Reference**

[1] Z. Li , B. Bai , F. Xie , Q. Zhao , “Distal Versus Total Gastrectomy for Middle and Lower‐Third Gastric Cancer: A Systematic Review and Meta‐Analysis,” International Journal of Surgery 53 (2018): 163–170, 10.1016/j.ijsu.2018.03.047.29602012

